# Up-Hill: An Allegory

**DOI:** 10.14797/mdcvj.1236

**Published:** 2023-05-16

**Authors:** James B. Young

**Affiliations:** 1 Emeritus Executive Director of Academic Affairs, Cleveland Clinic, US; 2 Professor Emeritus of Medicine, Cleveland Clinic Lerner College of Medicine of Case Western Reserve University, Cleveland, Ohio, US; 3 Section Editor, Poet’s Pen, Methodist DeBakey Cardiovascular Journal

## Abstract

The Christina Rossetti poem “Up-Hill” (1862) is an exemplary poem of the Victorian era by one of the rare female poets of the time, including Emily Brontë, Elizabeth Browning, Katherine Tynan, and Alice Meynell. Rossetti, typical of the era and the Victorian genre, wrote allegories about faith and love. She came from a distinguished literary family. “Up-Hill” was one of her better-known works.

## Up-Hill

Does the road wind up-hill all the way?

    Yes, to the very end.

Will the day’s journey take the whole long day?

    From morn to night, my friend.

But is there for the night a resting-place?

    A roof for when the slow dark hours begin.

May not the dark hide it from my face?

    You cannot miss that inn.

Shall I meet other wayfarers at night?

    Those who have gone before.

Then must I knock, or call when just in sight?

    They will not keep you standing at that door.

Shall I find comfort, travel-sore and weak?

    Of labour you shall find the sum.

Will there be beds for me and all who seek?

    Yea, beds for all who come.

Christina Rossetti

Originally published in *Macmillan’s*, February 1861

## Commentary: “Up-Hill”

Christina Rossetti (1830–1894) was a gifted poet from a gifted family. Her father was the Italian poet Gabriel Rossetti, who emigrated as a political exile to England before Christina’s birth. He was the chair of Italian Language at the newly opened King’s College, London.

The youngest of four children who were steeped in an academic environment, Christina was home schooled. She led a quiet life due to frequent bouts of strange illnesses, which may have been emotionally driven. She was engaged to one of the pre-Raphaelite Brotherhood painters, James Collinson, but did not marry him. The pre-Raphaelites were a band of English 19th century artists, poets, and writers who sought to emulate the simplicity and sincerity of the work of Italian artists before Raphael’s work.

Ultimately, Christina became one of the most respected Victorian Era poets. Though her poetry never really disappeared over time, its appreciation seems to have diminished a bit in recent years. However, interest in her work did expand significantly towards the end of the 20th century due to the emergence of “feminist criticism,” with its focus on the gender issues in her works and the fact that she was a woman poet, unusual for the time. *Poetry Foundation* notes that “In her lifetime opinion was divided over whether she or Elizabeth Barrett Browning was the greatest female poet of the era.”^1^ After Browning’s death in 1861, Christina’s brother, Dante Gabriel Rossetti (famous in his own right) noted that Christina was “the finest poet since Mrs. Browning, by a long way.” Christina succumbed in 1894 of metastatic breast cancer 2 years after a mastectomy that was performed in her own home.

“Up-Hill” is a short allegorical poem that exchanges questions with answers perhaps comparing life to an uphill journey. It appears to reflect Christina’s Anglo-Catholic religious beliefs. It acknowledges life’s struggles while representing the straight-laced Victorian age and, more generally, Christina’s religious values. Unspoken themes in the allegory may include aging, death and dying, disease and health, and suffering, among other concepts. The painful task at hand is “uphill” all the way, but there is a reward at the top—is this Heaven?^2^ There does seem to be a place for everyone.

Another way to read this poem is from the perspective of our care-giving workday, particularly the workday during the COVID pandemic when everything was topsy-turvy and intensivists and nurses, in particular, were bombarded with cases, death rates were high, and burnout was rampant. It could be comforting to read “Up-Hill” while putting our workdays into perspective. Yes, at the end of a grueling shift, the “travel sore” will find a bed and comfort. It is good reassurance to have around for those tough, on-call days and nights: “Yea, beds for all who come.”

**Figure 1:**
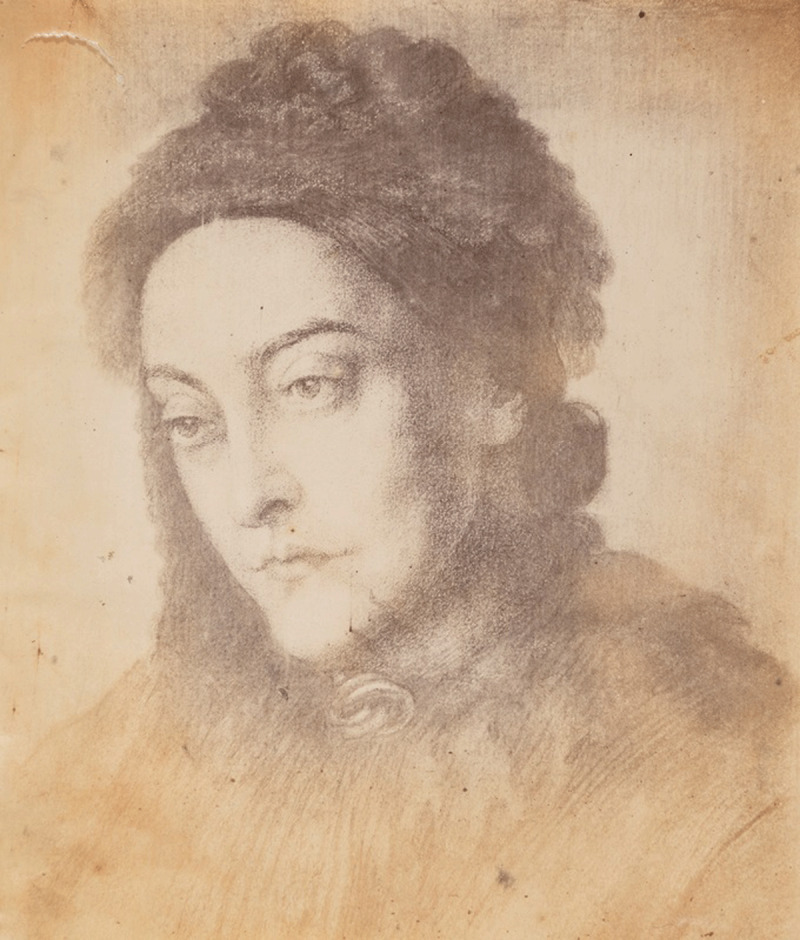
Portrait of Christina Rossetti painted by her brother, Dante Gabriel Rossetti. 2023 © Copyright National Portrait Gallery, London.
